# Modulation of the immune microenvironment using nanomaterials: a new strategy for tumor immunotherapy

**DOI:** 10.3389/fimmu.2025.1614640

**Published:** 2025-07-02

**Authors:** Haozhe Piao, Yuxin Jiang, Shengbo Jin, Jie Shi, Jun Yu, Wenping Wang, Zhenguang Du, Huini Yao, Qian Liu, Ningxin Li, Jiaqing Fu, Yue Shen, Mingzhu Li

**Affiliations:** ^1^ Department of Neurosurgery, Cancer Hospital of China Medical University, Liaoning Cancer Hospital & Institute, Shenyang, Liaoning, China; ^2^ Department of Integrated Traditional Chinese and Western Medicine Medical Oncology, Cancer Hospital of China Medical University, Liaoning Cancer Hospital & Institute, Shenyang, Liaoning, China; ^3^ Liaoning University of Traditional Chinese Medicine, Shenyang, Liaoning, China; ^4^ College of Acupuncture and Massage, Liaoning University of Traditional Chinese Medicine, Shenyang, Liaoning, China; ^5^ Dalian Medical University, Dalian, Liaoning, China; ^6^ Phase I Clinical Trial, Cancer Hospital of China Medical University, Liaoning Cancer Hospital & Institute, Shenyang, Liaoning, China; ^7^ Department of Interventional Medicine, Liaoning Provincial People’s Hospital, Shenyang, Liaoning, China; ^8^ China Medical University, Shenyang, Liaoning, China

**Keywords:** nanomaterials, tumor microenvironment, tumor immunity, cancer immunotherapy, application

## Abstract

The complexity of the tumor immune microenvironment (TIME), which is composed of mainly tumor cells, immune cells, and cytokines, is a major obstacle limiting the effectiveness of immunotherapy, and the interactions among these factors in the TIME determine the efficacy of antitumor immunity. Over the past few years, nanomaterials, owing to their unique physicochemical properties, multifunctionality, and good targeting ability, have gradually become important tools for modulating the immune microenvironment. By precisely delivering immunomodulatory factors, nanomaterials can effectively activate dendritic cells (DCs), enhance the function of effector T cells, and reverse the immunosuppressive state of tumor-associated macrophages (TAMs). In addition, nanomaterials can alleviate the local hypoxic and acidic tumor microenvironment, which in turn promotes immune cell function and enhances the antitumor immune effect. In light of the aforementioned associations, we summarize the existing studies, systematically describe the latest research progress on the use of nanomaterials in regulating the tumor immune microenvironment, and analyze the potential applications and challenges in tumor immunotherapy, with the goal of providing new therapeutic directions and strategies for tumor immunotherapy.

## 1 Introduction

Tumor immunotherapy, a major breakthrough in cancer treatment in recent years, works by activating the body’s immune defense system so that it can recognize and remove tumor cells. Immune cells constitute the cytological basis of immunotherapy. Therefore, understanding immune infiltration in the tumor immune microenvironment (TIME) is key to improving the potency of immunotherapy and developing new immunotherapeutic approaches (1). The tumor microenvironment is defined by cytokines and chemokines that facilitate immune suppression and an inflammatory condition. TME influences tumor differentiation, spread, and immune evasion. The TIME is a part of the tumor microenvironment (TME), and the TIME changes dynamically; as the tumor progresses, the immune system shifts from an immunosurveillance state to an immunosuppressive state. Initially, immune cells in the immune microenvironment attempt to attack tumor cells. On the other hand, as the cancer progresses, it alters the microenvironment, thereby suppressing the immune response (2).Nanoparticles possess unique properties (3, 4), and their precise delivery and targeting abilities and modifiability have made them important tools for modulating the TIME. These properties enable nanoparticles to deliver therapeutics directly to specific immune cells at the tumor site or in the tumor microenvironment, thus improving targeting. In addition, nanoparticles can be optimized to adjust the immune response, both by boosting antitumor immune cells and by suppressing immunosuppressive components of the tumor microenvironment (5). Not only do nanomaterials have many advantages, but nanomedicines are also advancing immuno-oncology research through their ability to deliver a variety of payloads and favorable molecular pharmacokinetics (6). The aforementioned benefits demonstrate that nanomaterials can effectively target drug delivery, influence immune cell activity, enhance drug delivery efficiency, and alter the tumor microenvironment. In recent years, the role of tumor-associated immune cells has led to new advances in tumor immunotherapy; thus, remodeling the TIME is a useful strategy for cancer treatment and immunotherapy (7, 8).

## 2 Characterization of the tumor immune microenvironment

The tumor microenvironment is a highly complex system consisting mainly of tumor cells, infiltrating immune cells, cancer-associated stromal cells, endothelial cells and adipocytes (9). In contrast, multiple immune cell populations in the tumor microenvironment, including inherent and adaptive immune cells, such as myeloid and lymphoid cells, constitute a major part of the TIME (10). In addition, other cell types, such as cancer-associated fibroblasts (CAFs), are also present in the TIME; the interactions between these cells and their functions together determine the role of the TIME in tumor progression (11, 12).

### 2.1 Cellular composition

The TIME consists of a complex network of multiple immune cell subtypes, cancer cells and stromal cells, which play key roles in tumor growth and disease progression (13). T lymphocytes, B lymphocytes, natural killer cells, dendritic cells, macrophages, myeloid-derived suppressor cells (MDSCs), fibroblasts and endothelial cells interact with tumor cells via sophisticated signaling pathways, forming a dynamic microenvironment (14, 15). The tumor immune microenvironment (TIME) is primarily composed of different immune cell populations found within the tumor microenvironment (TME), such as myeloid cells and lymphocytes, which include both innate and adaptive immune cells (16). IL-10 and analogous anti-inflammatory cytokines and chemokines can suppress the cytotoxic function of T cells and NK cells (17).

### 2.2 Immune escape

**Figure 1:**
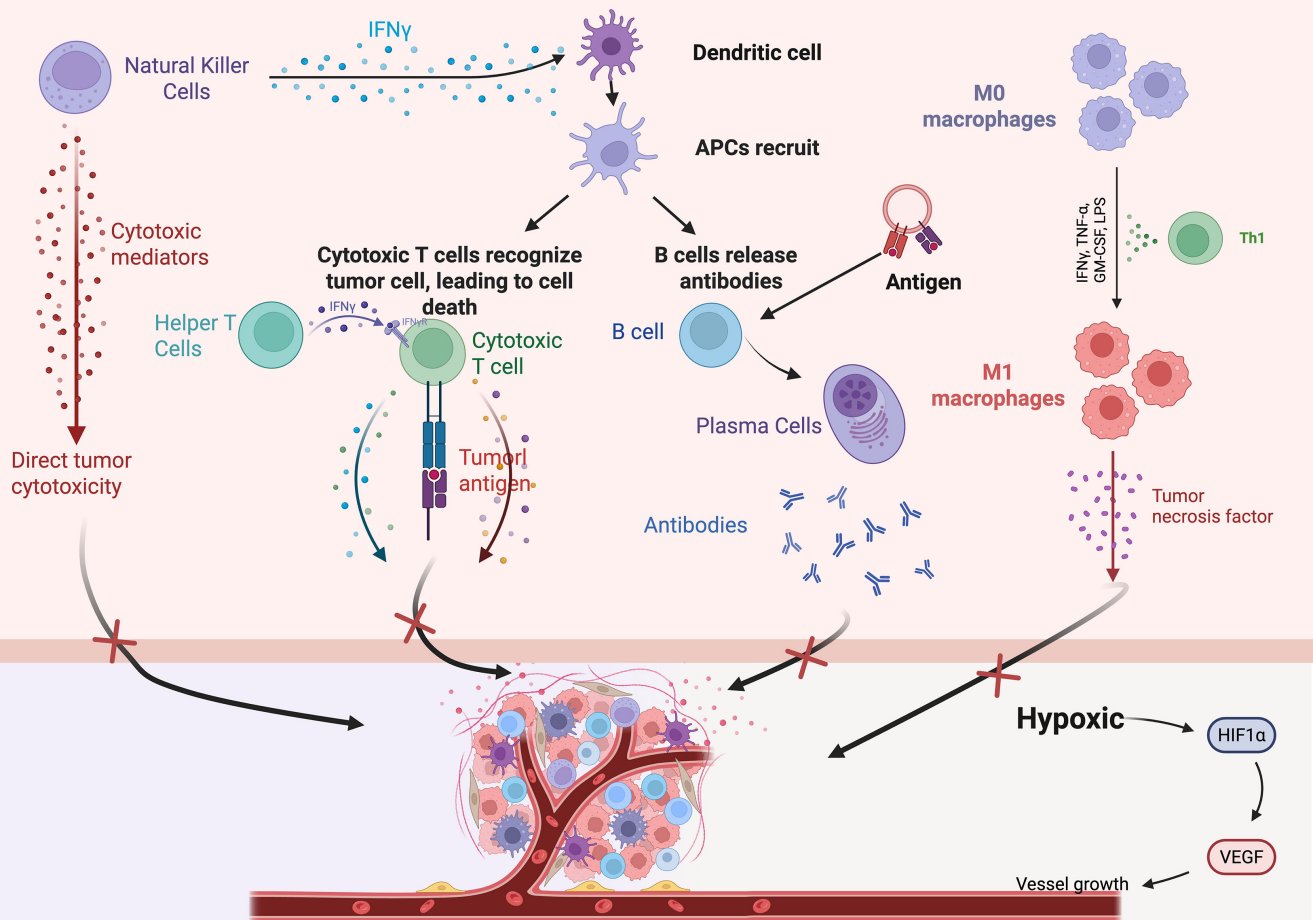
Schematic diagram of the tumor immune microenvironment, TIME major immune cells and their interactions. NK cells, helper T cells, IFNγ, cytotoxic T cells, DCs, etc.

The immune system shifts from a surveillance state to an immunosuppressive state as the tumor progresses (18). Tumor cells evade immune surveillance through a variety of mechanisms, which together result in the formation of an immunosuppressive tumor microenvironment. The main mechanisms are shown in **Table 1** (**Figure 1**).

**Table 1:** Mechanisms of tumor immune escape.

Mechanisms	Effects	References
Production of immunosuppressive factors	IL-10: IL-10 is a potent immunosuppressive factor that inhibits dendritic cell maturation and T-cell activity. TGF-β: TGF-β inhibits the multiplication and functionality of T cells, while promoting the differentiation and function of regulatory T cells. TGF-β production in the immunosuppressive microenvironment indirectly promotes tumor escape. Tumor cells escape immune surveillance by accumulating mutations that inhibit TGF-β signaling.	(123–125)
Immunosuppressive cell infiltration	Regulatory T cells (Tregs): Tregs are essential for the prevention of autoimmunity and have a suppressive effect on effector T cells. MDSCs: MDSCs inhibit specific and nonspecific T-cell responses by producing large amounts of nitric oxide, arginase-1, and immunosuppressive cytokines, such as IL-10.	(126, 127)
Upregulation of immune checkpoint molecules	PD-1/PDL-1: PD-1, as an immunosuppressive molecule, inhibits T-lymphocyte activation and promotes T-lymphocyte apoptosis. The abnormally high expression of PD-L1 in tumor cells is also considered to be one of the main factors promoting tumor immune escape.	(128–130)
Downregulation of antigen-presenting molecules	Tumor cells inhibit antigen presentation and evade immune surveillance by downregulating the expression of MHC molecules and antigen presentation-related molecules.	(131)

## 3 Tumor immunotherapy

Tumor immunotherapy is a therapeutic approach that mobilizes the body’s own immune system to attack tumor cells. In the last several years, immunotherapy has achieved remarkable advancements in the treatment of a wide range of cancers, making it one of the most important Strategies for the treatment of cancer.

Immune checkpoints, such as cytotoxic T-lymphocyte antigen-4 (CTLA4) and programmed cell death-1 (PD-1), are cell surface proteins that mainly control the initiation, duration and intensity of the immune response (19). The interaction between CTLA-4, PD-1 and PD-L1 increases the activity of protein phosphatase 2 and Src homology region 2 domain-containing phosphatases, inhibiting T-cell activity (20). In contrast, immune checkpoint blockade (ICB) therapy, targets T-cell regulatory pathways with immune checkpoint inhibitors to enhance antitumor immune responses, and this treatment has been shown to significantly improve patient survival compared with conventional cancer therapy (21). Chimeric antigen receptor T-cell (CAR-T) therapy is another type of cancer immunotherapy. Chimeric antigen receptor (CAR) refers to a synthetic receptor that enables lymphocytes to recognize and destroy cells expressing a specific target antigen. CAR activates T cells and enhances immune responses by binding to target antigens expressed on the cell surface independently of MHC receptors (22). However, CAR-T-cell therapy still has several limitations, including life-threatening CAR-T-cell-related toxicity, inhibition of malignant B cells, antigen escape, and poor durability (23). Therefore, there is an urgent need to identify new therapeutic means. In addition, tumor vaccines have been put into clinical use as a therapy to activate T cells and thus enhance immune responses. Currently, there are three main therapeutic tumor vaccines: cellular vaccines, peptide vaccines, and nucleic acid vaccines. The primary function of tumor vaccines is mainly to increase the infiltration of tumor-infiltrating lymphocytes in the tumor microenvironment or to increase their antitumor activity (24). However, some limitations in the clinical application of tumor vaccines remain. In addition, some cytokines in the tumor microenvironment such as interleukin-2 (IL-2), interferon-α (interferon-α, IFNα) and interferon-γ (interferon-γ, IFNγ) have also been used in immunotherapy approaches (25). Hypoxia and cytokine signaling pathways, which involve proinflammatory mediators such TNF-α and IFN-γ, are the main adaptive signaling pathways that upregulate immune checkpoint molecules (26).Consequently, it aids in preserving immunological tolerance and preventing assaults from the immune system. However, the clinical application of these therapies has also been hampered by their short duration and strong toxicity (27).

**Figure 2:**
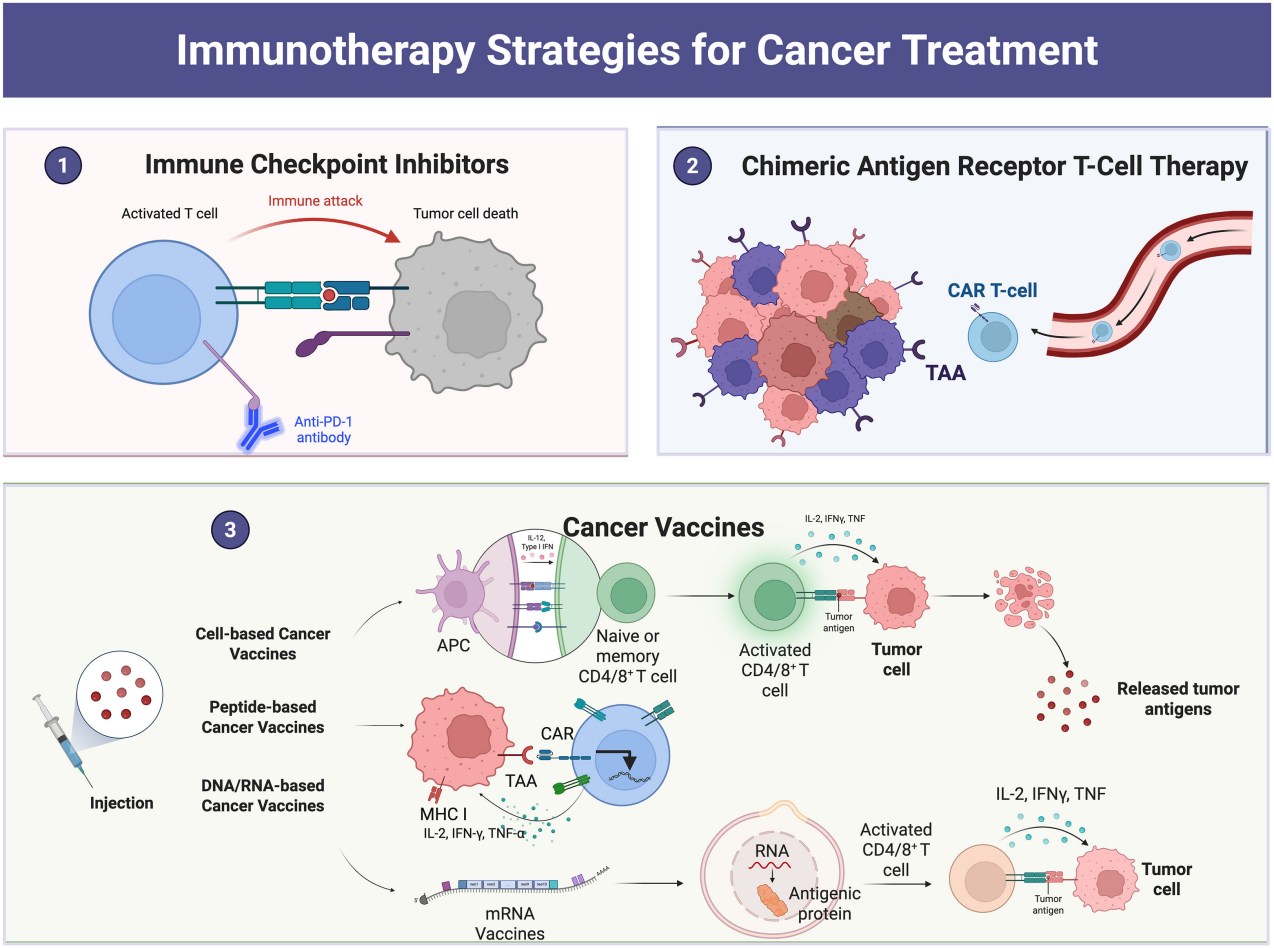
Examples of cancer treatment using immunotherapy: The figure shows immune checkpoint blockade therapy, CAR-T-cell therapy, tumor vaccines, etc.

Although the above therapies have certain therapeutic effects, they still have certain limitations and shortcomings, so the search for new therapeutic means is one of the urgent clinical problems to be solved (**Figure 2**).

## 4 Application of nanomaterials in modulating the tumor immune microenvironment

**Figure 3:**
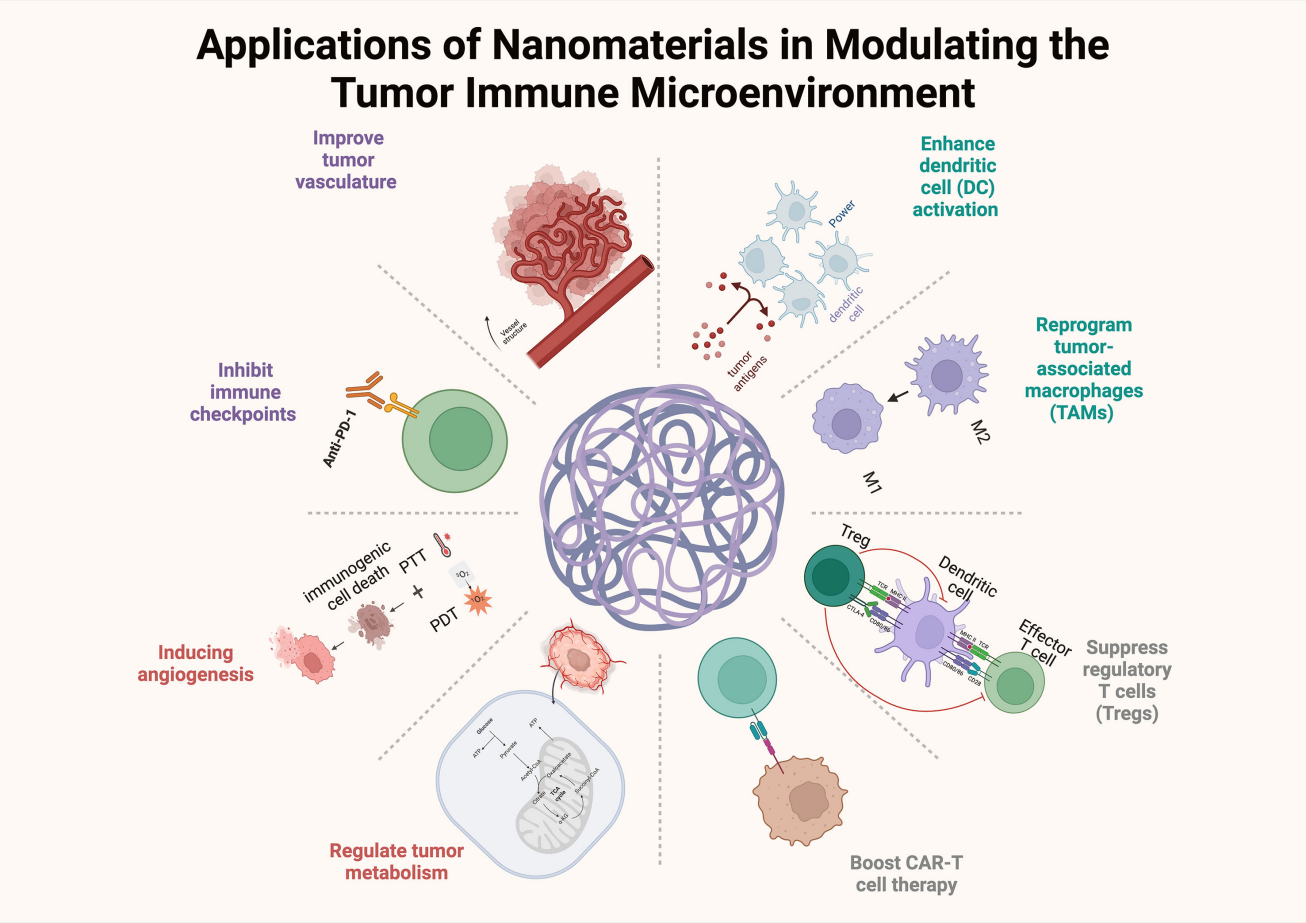
The effects of nanomaterials on the TIME, including the reprogramming of tumor-associated macrophages, inhibition of immune checkpoints, and CAR-T cell therapy.

Owing to their small size and excellent physical, chemical and biological properties, nanomaterials have good potential for applications in medicine, energy, environmental protection, and electronics (28). Nanomaterials can act on complex and variable environments in the TIME and can specifically bind to immunosuppressive cells, thus improving immunotherapy efficacy. In addition, drug delivery systems constructed with nanoparticles have become favorable strategies for cancer treatment; for example, the main advantage of the utilization of nanoparticles as vehicles for drugs is that they can encapsulate the drug and transport it directly to tumor cells, which not only reduces drug toxicity but also maximizes the efficacy of the drug. Therefore, nanoparticles can be specifically developed to target components of the tumor microenvironment (TME) and disrupt the immunosuppressive TME, thereby enhancing the efficacy of cancer immunotherapy. Nanomaterials are categorized into organic nanomaterials, inorganic nanomaterials, and composite nanomaterials according to their composition (29). Therefore, in this work, we describe the regulatory effects of the above three different types of nanomaterials on the TIME in detail and analyze the potential application of these nanomaterials in immunotherapy (**Figure 3**).

### 4.1 Organic nanomaterials

**Figure 4:**
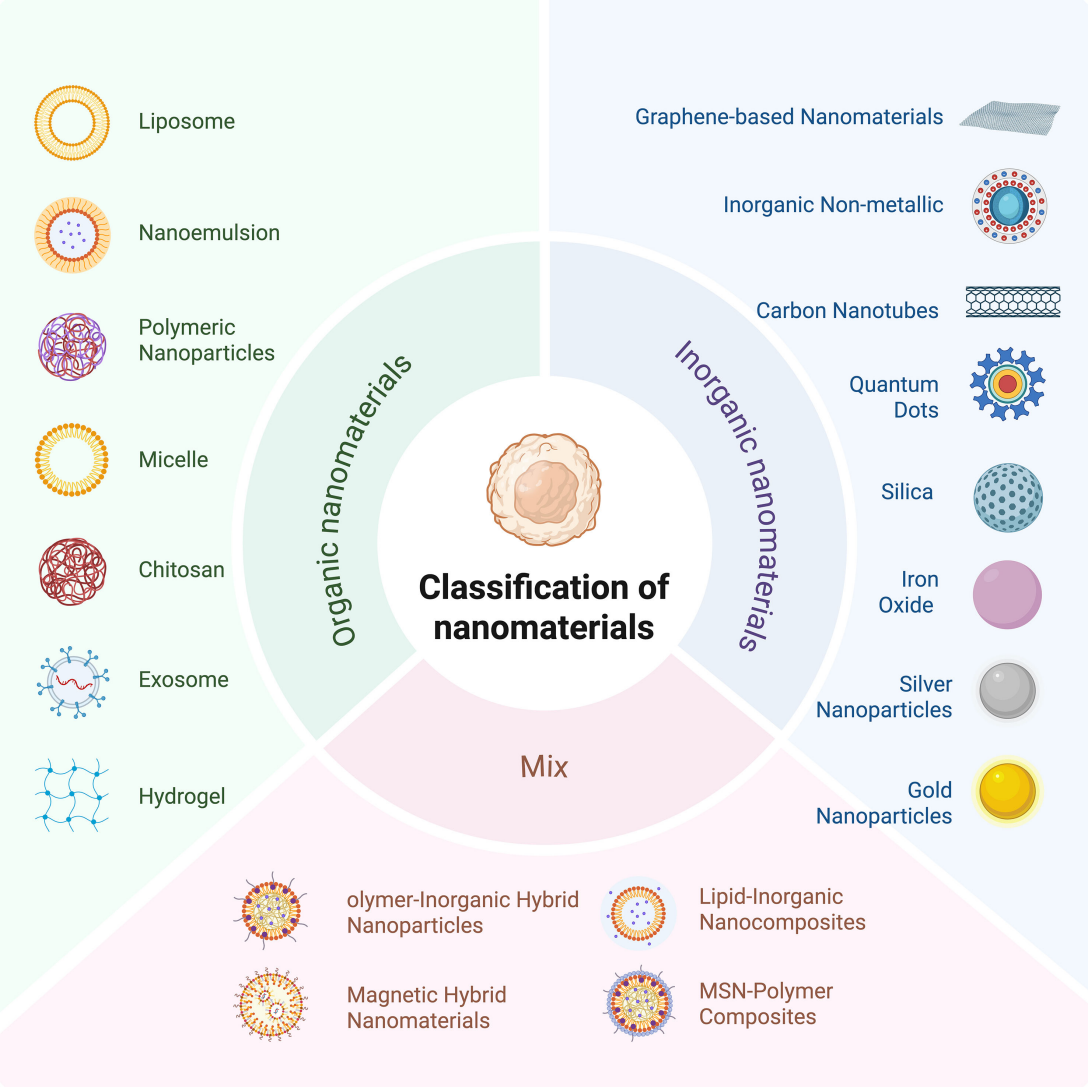
Classification of nanomaterials into organic nanomaterials, inorganic materials, and composite nanomaterials according to their composition.

Organic nanomaterials usually consist of naturally occurring organisms or compounds. Compared with inorganic materials, they are less cytotoxic and biodegradable (30). Organic nanomaterials are mainly composed of carbon, hydrogen, oxygen, and nitrogen; the biosafety of organic nanoparticles is a prominent advantage, and their biodegradability mitigates their accumulation in the body (31). Common types include polymer nanoparticles, liposomes, and dendrimers (**Figure 4**).

#### 4.1.1 Liposomes

Liposomes consist of sterols, surfactants and natural or synthetic phospholipids, such as those that can be obtained from egg yolk, soybean and hydrogenated phosphatidylcholine, which are degradable, nontoxic and suitable for industrial production (32). In addition, liposomes are spherical vesicles with bilayer membranes (33). These bilayers are spontaneously formed by phospholipids dispersed in an aqueous phase medium, and the amphiphilic nature of liposomes makes them effective drug carriers (34) and significantly improves the transport of drugs across various lipid-based barriers (35). In addition, liposomes can improve the therapeutic efficacy of drugs by increasing drug solubility and reducing drug toxicity (36).

Liposomes have been used in a variety of therapeutic applications, such as anticancer drugs, antifungal drugs, antibiotics, gene therapy, anesthetic drugs, and anti-inflammatory drugs. Previous studies have revealed the ability of nanodrug delivery systems to modulate the tumor immune microenvironment and enhance the antitumor immune response (37, 38). Gu et al. (39)summarized the many advantages of liposomes as carriers, including their use as vaccines that stimulate immune responses, their ability to selectively deliver drugs to the tumor microenvironment, and their ability to be used in conjunction with other therapies, including chemotherapy, radiotherapy and photothermal therapy. Cheng et al. (40)mixed hydrogenated (soybean) L-α-phosphatidylcholine and 1,2-dioleoyl-3-trimethylammonium propane in a 100:1 weight ratio, dissolved them in chloroform and dried them with a rotary evaporator to form a lipid film. The liposomal nanoparticles were able to deliver cGAMP to macrophages via STING receptors in triple-negative breast cancer. cGAMP reprogrammed M2-like macrophages in the TME to M1-like macrophages and increased CD4^+^ and CD8^+^ T-cell infiltration, thereby enhancing the efficacy of breast cancer immunotherapy. In addition, many factors in the TME, such as temperature and pH, can influence the action of the TME; therefore, Fu et al. (41) developed a temperature-sensitive liposome-based delivery system, which was able to remodel the immune microenvironmental of tumor lymph nodes and induce immunogenic cell death in tumor cells after stimulation by photothermal therapy, thus increasing the levels of calmodulin and the secretion of high-mobility protein B1 to promote dendritic cell maturation. Fu et al. (41) also demonstrated strong antitumor immunotherapy against hot and cold tumors when used in combination with PD-L1 therapy. In addition to temperature-sensitive liposomes, pH-sensitive liposomes have been used for similar functions. CAFs are prevalent and engage with cancer cells and the adjacent tumor microenvironment (TME), significantly influencing tumor development, proliferation, and metastasis (42). Moreover, CAFs restructure the extracellular matrix by the secretion of growth factors, chemokines, and other tumor-enhancing substances (43). Consequently, there is a growing emphasis on CAFs and their integration with nanomaterials to modify the tumor microenvironment and improve the effectiveness of immunotherapy. Jia et al. (44) fabricated liposomes encapsulated in CAFs and tumor cell membranes that were able to specifically target CAFs and tumor cells. In addition to drug delivery, liposomal vaccines have shown great potential; a previous study revealed that a liposomal vaccine consisting of the cationic lipid DOTAP and loaded with mRNA was able to induce an immune response against melanoma (45).

#### 4.1.2 Lipid nanoparticles

Lipid nanoparticles (LNPs) are colloidal nanocarriers ranging from 1–100 nm in size. Compared with metal nanoparticles, LNPs are more easily removed and accumulate less in the human body; thus, LNPs are less toxic to organisms (46).

LNPs have been extensively studied for use in small interfering RNA (siRNA) and messenger RNA (mRNA) delivery. SiRNA largely addresses cancer by silencing genes, which is essential for tumor development. Among siRNA delivery techniques, liposomal nanoparticles are the most thoroughly investigated. Most siRNA nanoparticle delivery strategies depend on the increased permeability and retention (EPR) effect to increase tumor accumulation. The EPR effect can enhance the diffusion of siRNA nanoparticles to several organs (47).

**Figure 5:**
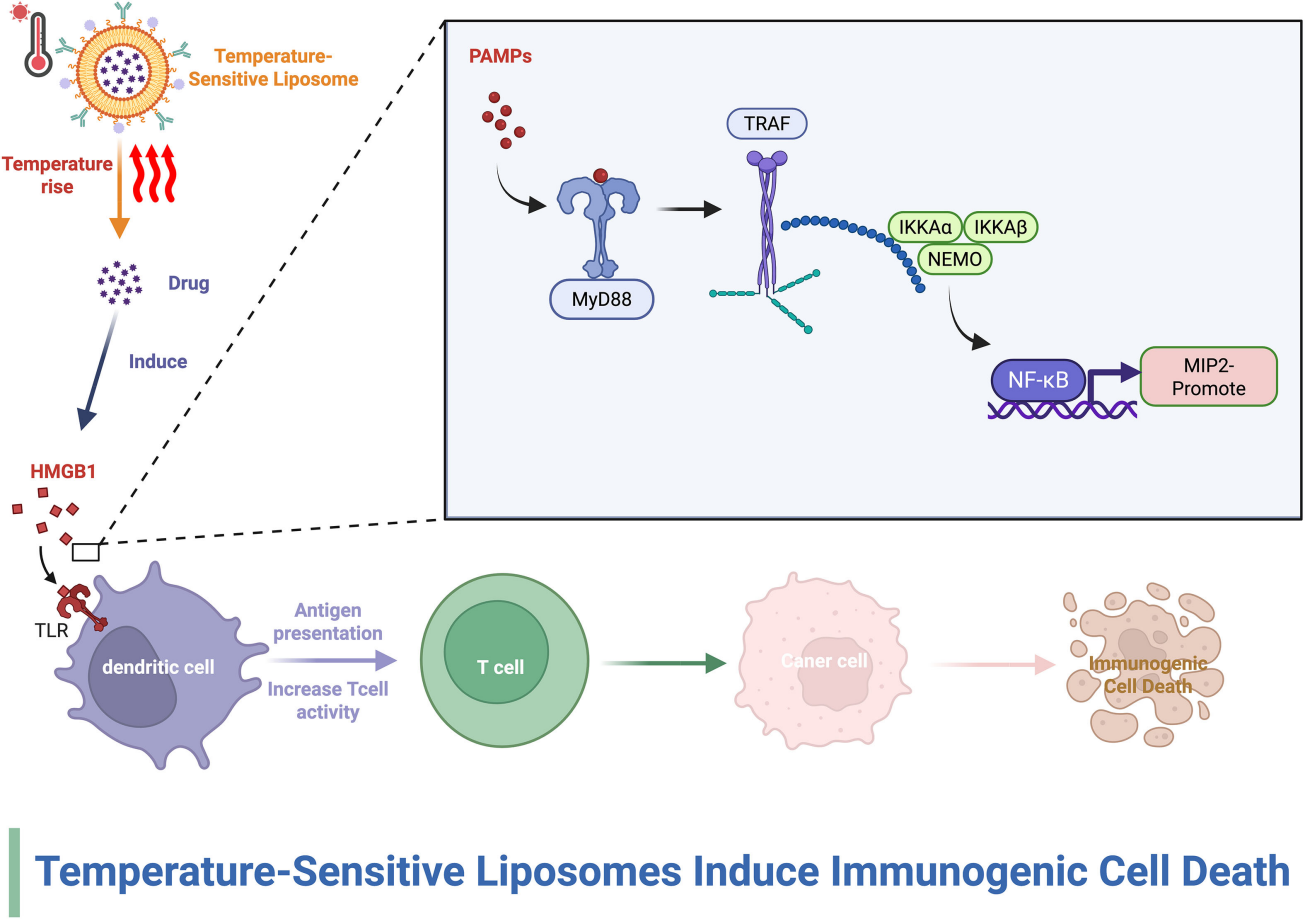
The effect of liposomes on the TIME. This figure demonstrates that thermosensitive liposomes release medicines in response to hyperthermia, thereby causing immunogenic cell death (ICD) in tumor cells.

Compared with cationic liposomes, these lipids are neutral at physiological pH, are protonated at low pH, and have the advantages of greater biocompatibility and lower cytotoxicity (48). CRISPR/Cas is a gene editing technology, the RNA and protein constituents of the CRISPR/Cas9 system are prone to degradation in the *in vivo* milieu. Lipid nanoparticles (LNPs) safeguard these components against degradation by nucleases and proteases via their lipid shell, thus extending their stability within the body. Altering the surface of lipid nanoparticles (LNPs) can diminish their identification by the immune system, hence reducing the immunogenicity of the CRISPR/Cas9 system *in vivo*. It has been shown that by embedding the CRISPR/Cas system into the LNP system, nanocarriers can be target and deliver the CRISPR/Cas system to cancerous tissues; moreover, by incorporating specific antibodies, nanocarriers can reduce off-target effects and enhance their targeting ability (49). Mennati et al. (50) prepared heterotrimeric LNPs loaded with siRNA and lycopene and demonstrated that blockade of the IGF-1R signaling pathway could inhibit cell proliferation and lead to programmed cell death and that LNPs significantly induced apoptosis and blocked the cell cycle in MCF-7 breast cancer cells. Zhang et al. (51) formulated an mRNA vaccine utilizing lipid analogs and designed and screened specific LNPs that activated Toll-like receptor 4 and induced T-cell activation; the results showed that the nanovaccine could deliver mRNAs to the DCs and promote antigen presentation; in addition, LNPs also have good immunogenicity and can induce T-cell activation, thus improving the immunotherapeutic effect on tumors (**Figure 5**).

#### 4.1.3 Dendritic macromolecules

Dendritic macromolecules are nanoscale molecules with radial symmetry as well as well-defined, homogeneous, monodisperse structures. Dendritic polymers are usually between 4 and 20 nm in diameter, which is smaller than most nanoparticles and liposomes; this feature makes them potentially more advantageous in terms of interstitial diffusivity, cellular uptake volume and tissue penetration depth (52). Dendrimers are usually used as nucleic acid carriers, and in the treatment of cancer, nucleic acid therapy has a great advantage (53).

Zhan et al. (54) designed a nanomicelle composed of a phosphorus dendrimer and the chemotherapeutic drug doxorubicin, and the material combined with aPD-L1 treatment inhibited tumor growth, enhanced the apoptosis of B16 cells, induced immunogenic cell death, and promoted the proliferation and activation of natural killer cells, which in turn affected the TIME in a mouse melanoma model. Xiang et al. (55) developed an amphiphilic PAMAM G5 dendrimer that enhances the pH sensitivity of drugs in the TME. While Huang et al. (56) also developed the above dendrimer material and found that it was able to release Fe^3+^ and Cu^2+^ in a weakly acidic TME. Moreover, the targeted MR imaging of triple-negative breast cancer was achieved by modulating the TME with enhanced iron death and chemodynamic therapy. The TME contains a high concentration of glutathione (GSH), and glutathione enzymes can participate in the development of chemotherapeutic resistance, thus reducing the efficacy of conventional chemotherapeutic drugs (57). Zhang et al. (58) developed a smart nanomedicine formulation based on redox-responsive dendritic macromolecular nanogels, which not only has good biocompatibility and the ability to reduce the high concentration of GSH in the TME but also has the ability to activate tumor-infiltrating CD 4^+^ and CD 8^+^ T cells, in addition to being able to resist PD-L1 immune checkpoint blockade therapy, remodel the TIME and inhibit tumor growth.

#### 4.1.4 Exosomes

**Figure 6:**
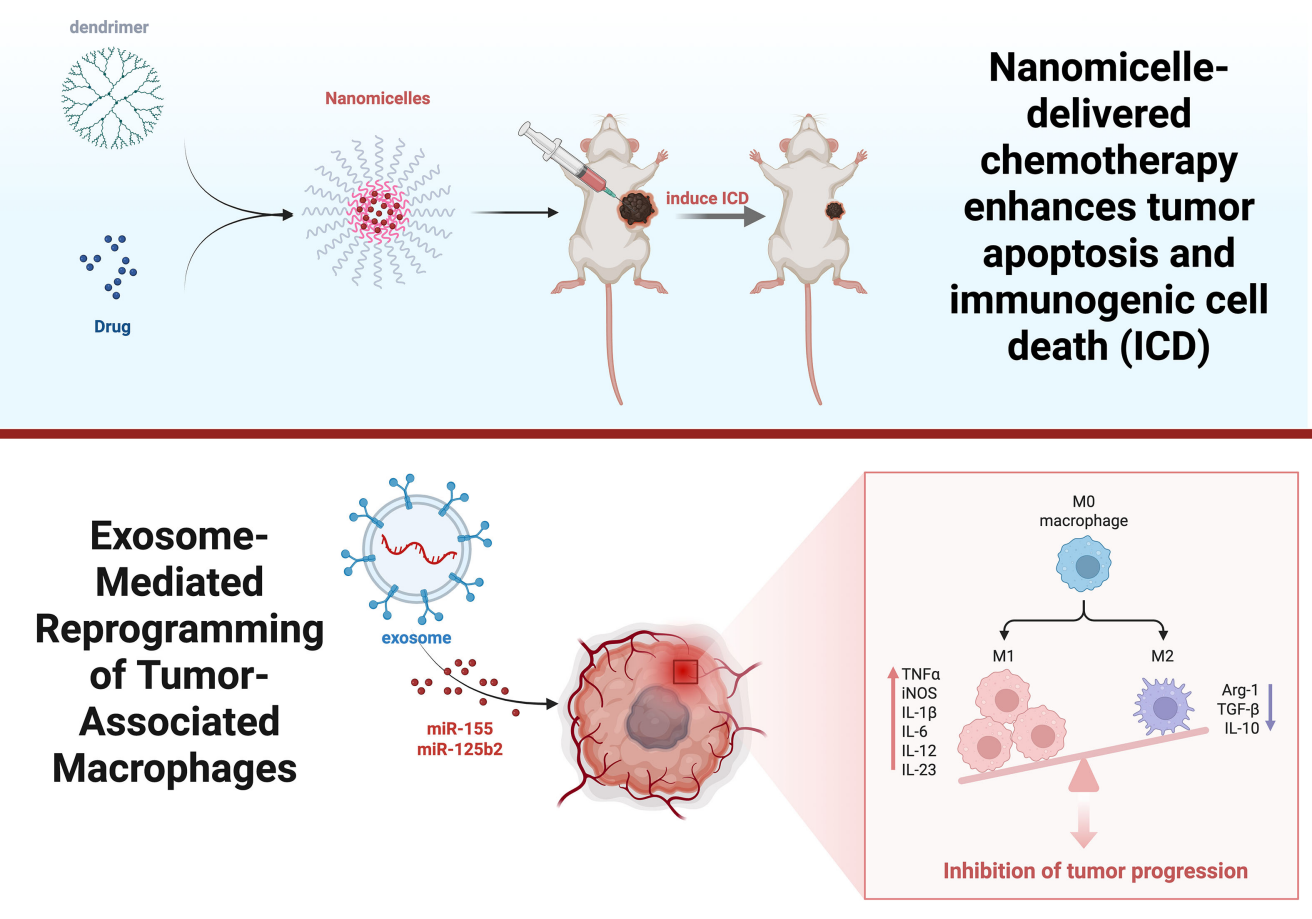
Effects of dendrimer macromolecules and exosomes on the TIME. This picture clarifies how dendrimers and nanomicelles facilitate ICD by drug delivery, subsequently activating anticancer immune responses. Exosomes containing miRNAs reprogram TAMs.

Exosomes, also known as extracellular vesicles, are the smallest organelles, with a size of approximately 40–150 nm (59). Exosomes play important roles in human growth and development, immunomodulation, and tissue homeostasis (60), and they serve as versatile drug delivery vehicles that can be used to regulate immunity (61). In addition, although exosomes are similar to liposomes in shape and function, compared with liposomes, exosomes are retained in body fluids for a longer period of time and are not easily eliminated by macrophages or reticuloendothelial cells (62). In recent years, the application of exosomes in tumor immunity has become increasingly widespread; relevant studies have shown that miR-155 and miR-125b2 in exosomes can be used to reprogram tumor-associated macrophages, thereby inhibiting tumor progression (63). Fu et al. reported that exosomes derived from DCs can be loaded with a variety of peptide antigens and that they stimulate both CD4^+^ and CD8^+^ T cells, which participate in antitumor responses (64). M1-like macrophage-derived exosomes (M1-Exos) have inflammation-directed tumor-targeting ability, and relevant studies have shown their role in reprogramming the immunosuppressive TME (65). Zhen (66) developed an exosome bound to liposomes that could effectively alleviate hypoxia, increase ROS levels, promote the release of proinflammatory cytokines from M1-Exos and reprogram the immunosuppressive TME. Lv et al. (65) also developed a nanovesicle composed of exosomes and AS1411 aptamer-modified liposomes, which not only generate a large amount of reactive oxygen species to induce the apoptosis of tumor cells via near-infrared laser irradiation but also polarize TAMs and promote the infiltration of T lymphocytes, thus enhancing the antitumor immune response (**Figure 6**).

#### 4.1.5 Nanogels

Nanogels can be used as nanocarriers for systemic drug delivery. Nanogels take advantage of the combination of nanotechnology with gel-based materials such as hydrogels (67). Nanogels have unique physical and chemical properties, such as nanoscale size, tunable properties, significant hydration, good biocompatibility, etc. (68). Compared with conventional nanoparticles, they have variable particle sizes and shapes as well as greater sensitivity to external stimuli such as pH, temperature, ionic strength and redox conditions (69).

Bai’s team (70) developed a nanogel that responds to matrix metalloproteinase-2 in the TME and releases two types of liposomes at the tumor site; it not only promotes apoptosis but also triggers immunogenic cell death and promotes the maturation of DCs and T-cell infiltration, thus altering the immune-suppressive state of the TME and enhancing the therapeutic effect. In contrast, Li et al. (71) designed a biocompatible alginate-based hydrogel for codelivery of dextran nanoparticles encapsulated with pessidatinib and platelets modified with an anti-PD-1 antibody; this material was able to inhibit macrophage aggregation, significantly alleviate the immunosuppressive tumor microenvironment, promote infiltration of effector CD8^+^ T cells, and enhance the immunotherapeutic effect of tumors by depleting TAMs. In addition, Jiang et al. (72) developed an injectable hydrogel for the delivery of macrophage CAR gene-loaded nanocarriers and anti-CD47 antibodies capable of targeting glioma stem cells and triggering an antitumour immune response. Tian et al. (73) prepared a self-degradable nanogel that efficiently facilitated the infiltration and activation of CD8^+^ T cells and remodeled the immunosuppressive TME. Gao et al. (74) developed Vir-Gel, a membrane-encapsulated nucleic acid nanogel embedded with therapeutic miRNAs, which could induce the calibration of proinvasive M2 macrophages to antitumor M1 macrophages and enhance the immunotherapeutic efficacy in glioma.

#### 4.1.6 Polymeric nanoparticles

Polymeric nanoparticles (PNPs) are entities whose size falls within the range of 1 to 1000 nm, mainly including nanospheres, nanocapsules, and polymeric micelles (75). Polymeric nanoparticles are usually characterized by good biocompatibility, modifiability, etc., and can be either derived from nature or artificially synthesized. Chitosan, as a type of polymer nanoparticle, is derived mainly from the deacetylated form of chitin; it is one of the best drug carriers for the treatment of cancer (76). Micelles are nanocolloidal particles formed by the assembly of amphiphilic polymers. Targeting CAFs is one strategy in tumor immunotherapy, and Cheng et al. (77) demonstrated that polymeric micelles can noncovalently bind CAF-targeted antibody fragments, thus improving the efficacy of immunotherapy. Wang et al. (78) integrated paclitaxel, thioridazine and the small-molecule PD-1/PD-L1 inhibitor HY19991 into a dual-enzymatic pH-sensitive polymer structure that was able to release paclitaxel in the acidic TME. This approach was almost always able to alleviate the immunosuppressive TME in a mouse model of MCF-7 metastatic breast cancer (79). In addition, Wan (80) synthesized a micellar system assembled with gemcitabine-conjugated polymer (PGEM), a CCR2 antagonist PF-6309, for cancer therapy; PGEM was shown to activate the STING signaling pathway in DCs, which enhanced natural killer cell and adaptive antitumor T-cell responses and reduced the aggregation of TAMs. Groettrup et al. (81) created a new type of cancer nanovaccine using PLGA and riboxxim. This vaccine can activate the immune response by triggering endosomal Toll-like receptor 3. Dacoba et al. (82) designed nanocomplexes capable of targeting and delivering the Toll-like receptor 3 agonist poly(I:C), and *in vitro* experiments revealed that poly(I:C) delivered by this nanocarrier was more efficiently internalized by macrophages, which facilitated macrophage polarization toward the M1 phenotype, thereby enhancing their antitumor effects.

**Figure 7:**
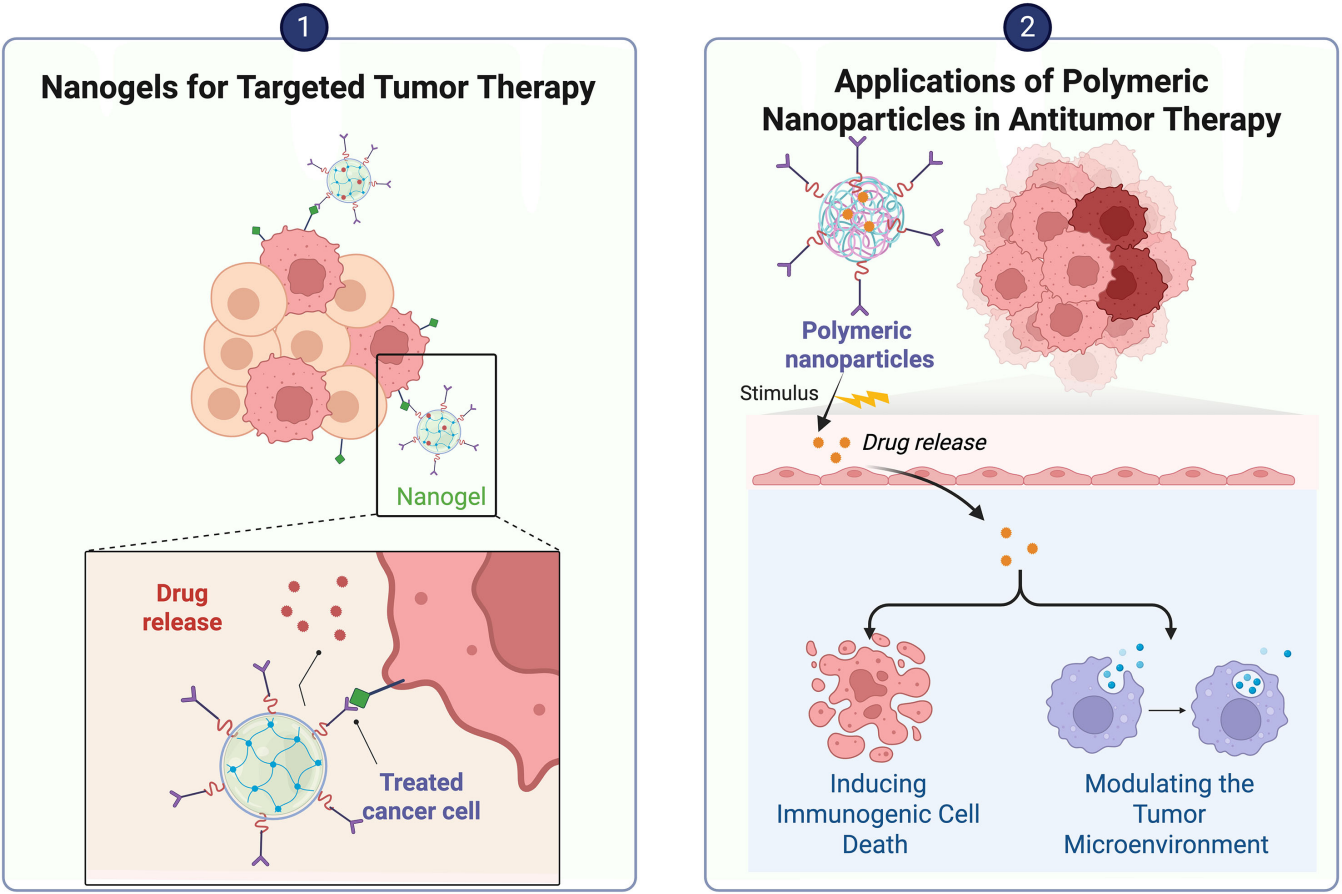
The mechanisms by which hydrogels and polymer nanoparticles deliver drugs to the TIME.

Guo et al. (83) prepared organic complex nanoparticles with photothermal capability, which, while being controllable for photothermal therapy and reducing damage to the surrounding tissues, were also able to significantly increase the infiltration of CD4^+^ and CD8^+^ T cells in the TME, downregulate the expression of PD-L1, and increase the proliferation and activity of cytotoxic T lymphocytes, thus improving the antitumor immune response of the organism (See **Table 2**) (**Figure 7**).

**Table 2:** Functions and advantages of organic nanomaterials in the TIME.

Name	Function in TIME	Advantage	References
Liposomes	Increase the infiltration of CD4+ T cells and CD8+ T cells in tumor tissues Decrease the infiltration of Tregs in tumor tissues and their immunosuppressive effects	Liposomes can protect drugs from degradation by the physiological environment, thus prolonging the half-life of drugs Through surface modifications, such as PEGylation or additional targeting ligands, liposomes enable the specific targeting of tumor cells Liposomes have the ability to control drug release.	(41, 132, 133)
Dendrimers	Immunomodulation: Promote proliferation and recruitment of natural killer cells and cytotoxic T lymphocytes	Good stability and pH-responsive drug release properties	(54)
Exosomes	Suppress the growth and invasion of tumor cells (B16) Modulate the polarization status of immune cells, especially macrophages, in the tumor microenvironment by increasing the proportion of M1-type macrophages	Low toxicity Efficient targeting ability Enhance the properties of tumor stem cells Regulate the function of immune cells	(134–137)
Nanogels	Increase immune cell infiltration Improve drug delivery	Biocompatibility: Nanogels are usually made of biocompatible materials. Drug loading capacity: Nanogels have high drug loading capacity.	(138, 139)
Polymer nanoparticles	Regulates the function of immune cells: can carry immune checkpoint inhibitors, cytokines, etc.Can prolong drug action according to conditions in TIME	Can load drugs Strong stability	(140, 141)

### 4.2 Inorganic nanomaterials

Inorganic nanomaterials not only have controllable shapes and sizes but also well-defined chemical properties and excellent optical, electrical, and magnetic properties, thus showing great advantages in tumor immunotherapy (84, 85). The research and application of inorganic non-metallic biomaterials cover a wide range of fields. In addition to tissue engineering, they also include the diagnosis and treatment of diseases and drugs, such as imaging technology, tumor radiosensitization treatment, immune regulation, sterilization, etc. The inorganic non-metallic materials mainly used in these aspects are at the nanoscale (86). Inorganic nanomaterials are mainly classified into metallic and nonmetallic nanomaterials.

#### 4.2.1 Metallic nanoparticles

Metal ions are vital to tumor immunomodulation, and some scholars have proposed the use of cancer metal immunotherapy (87). Combining metal ions with nanomaterials and preparing corresponding metal nanoparticles can modulate the immune response to tumors. Metallic nanoparticles can be designed to enhance biocompatibility and reduce toxicity to normal tissues by modifying their surface. In addition, metallic nanoparticles can act on tumor cells, immune cells and the extracellular matrix simultaneously to modulate the TME and enhance therapeutic effects.

Among many metal nanoparticles, gold nanoparticles are widely used because of their good biocompatibility and stability (88). Gold nanoparticles can induce an effective immune response at low doses (89). Liang et al. (90) subcutaneously injected gold nanomaterials encapsulated in liposomes into tumor-bearing mice, and 12 hours after the injection, the composite nanoparticles reached maximum accumulation in local lymph nodes and significantly promoted the maturation of dendritic cells, indicating that gold nanomaterials can be used to activate immune cells and enhance immune responses.

In contrast, among inorganic nanomaterials, only iron-based nanoparticles are approved by the FDA for medical use (91). Iron-based nanomaterials can enhance the killing effect on tumor cells through the Fenton reaction (92). Zhang et al. (93) developed nanomaterials consisting of Fe/Cu-based metal–organic frameworks, lactic acid oxidase and hyaluronic acid, which were able to increase the level of intracellular reactive oxygen species (ROS) via the glycolysis, which then promoted the repolarization of TAMs and enhanced the efficacy of immunotherapy.

CAFs inhibit the activity of immune cells by secreting immunomodulatory factors, which in turn promote the accumulation of immunosuppressive cells, such as Tregs and MDSCs, thereby helping tumor cells evade immune surveillance (94). Therefore, Bromma et al. (95) assayed gold nanoparticles modified with polyethylene glycol and arginine-glycine-aspartic acid peptides on the surface inside tumor cells and reported that the nanoparticles were able to increase cellular uptake and significantly reduce the tumor volume. In photodynamic therapy (PDT), photosensitizers are used to generate reactive oxygen species (ROS) under specific light irradiation, which is able to induce tumor cell death and amplify the efficacy of tumor immunotherapy (96). Zhou et al. (97) prepared ferritin nanoparticles, which are able to deliver photosensitizers to CAFs, significantly increase their photosensitizer accumulation at the tumor site, and also generate an immune response toward CAFs, thus enhancing the effect of immunotherapy. Ding et al. (98) formulated a vaccine (ZPM@OVA-CpG) that was able to release Zn^2+^ in a controlled manner and improve immunotherapy efficacy. Deng et al. (99) demonstrated the ability of a calcium-manganese bionic hybrid nanostimulant to activate both apoptosis and the innate immune response in ferrocytes, which provided a new therapeutic idea for the effective immunotherapy of triple-negative breast cancer. Cen et al. developed a functional immunotherapy for triple-negative breast cancer.

#### 4.2.2 Metal oxide nanoparticles

Metal oxide nanomaterials are nanoscale materials composed of metal and oxygen, mainly zinc oxide, iron oxide and other nanomaterials. Sun et al (100). prepared a hyaluronic acid-optimized magnetic nanoparticles (Fe_3_O_4_), which can not only efficiently penetrate into the interior of tumors but also adhere to CD44 receptors on the surface of tumor cells via hyaluronic acid to achieve specific targeting of tumor cells. In addition, these nanoparticles can regulate TAM repolarization and stimulate tumor cells and macrophages to secrete more chemokines. In contrast, Yang’s team (101) engineered a novel biomimetic magnetic nanoparticle, Fe_3_O_4_-SAS@PLT, which not only elicits tumor-specific immune responses but also effectively repolarizes macrophages from the immunosuppressive M2 phenotype to the antitumor M1 phenotype, in addition to depleting glutathione (GSH).

**Figure 8:**
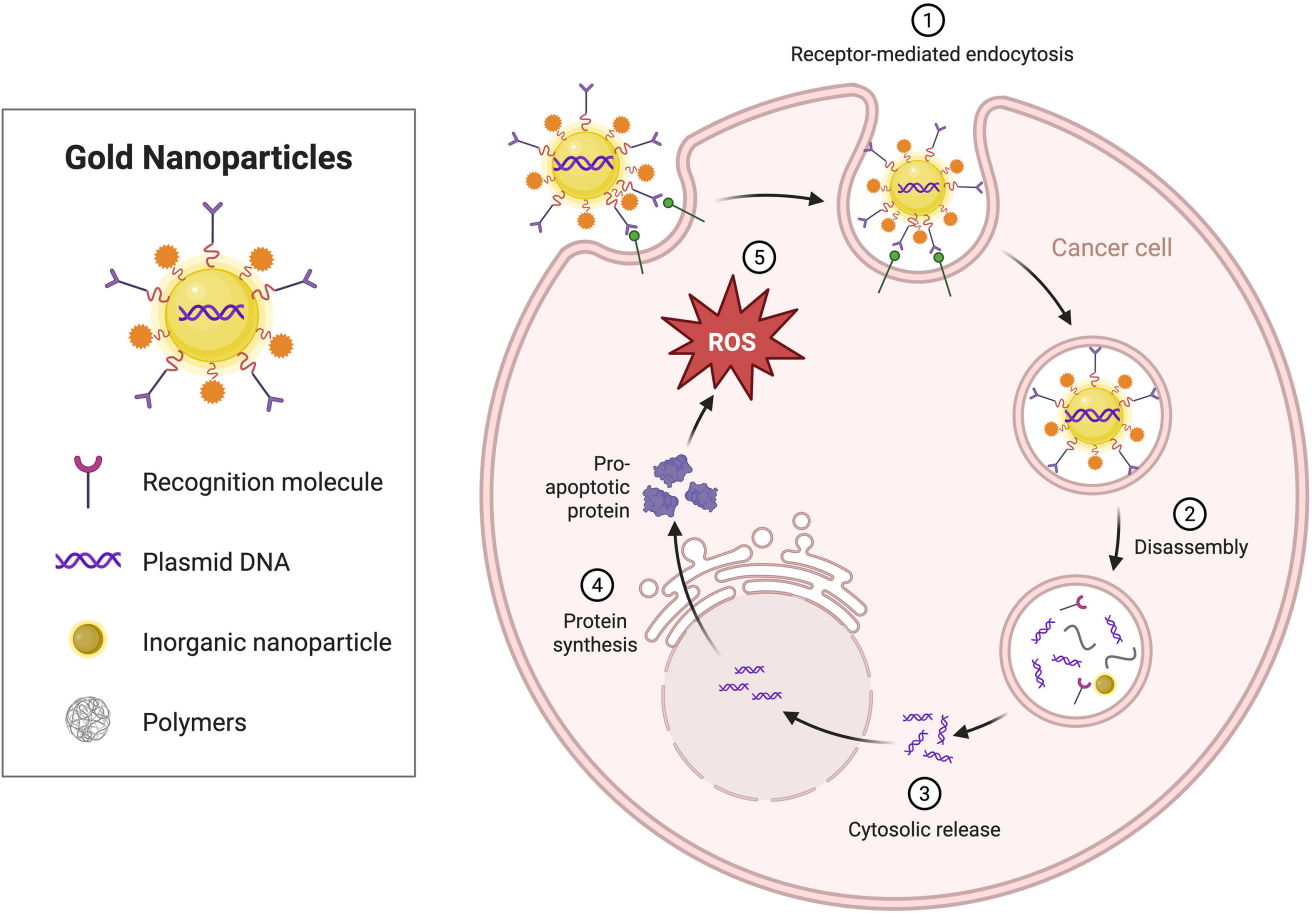
The effect of metallic nanomaterials on the TIME. Gold nanoparticles are modified on their surfaces with recognition molecules, such as plasmid DNA and polymers, enabling their entry into cancer cells via receptor-mediated endocytosis.

ROS can modulate the TIME and promote the infiltration and activation of immune cells, thereby enhancing antitumor responses. ROS usually act as intermediate signaling molecules, affecting the differentiation, function, and secretion of TAMs (102). Previous studies have shown that ROS-scavenging nanozymes can inhibit the transformation of macrophages into M2 TAMs by suppressing the activity of the ERK and STAT3 signaling pathways (103). Therefore, Gong et al (104). used bimetallic oxide FeWOX nanosheets in combination with a CTLA-4 checkpoint blocker and reported that ROS-mediated inflammation induced by FeWOX nanosheets in the TME can activate the immune system and trigger a strong immune response, which not only promotes the maturation of DCs, increases the number of T cells, and decreases the number of Tregs but also induces the repolarization of TAMs, thus effectively inhibiting tumor growth. Wu et al. (105) prepared a composite metal oxide nanomaterial of titanium carbide-chitosan-manganese iron oxide (MnFe_2_O_4_) that was able to catalyze the oxidation of glutathione to oxidized glutathione, thus depleting excess glutathione in the TME and increasing ROS generation and therapeutic effects (**Figure 8**).

#### 4.2.3 Nonmetallic nanomaterials

Non-metallic nanoparticles are particles at the nanoscale composed of non-metallic elements or non-metallic compounds. Graphene oxide nanosheets prepared by Guo et al. (106) were not only able to stimulate the polarization of M1-type macrophages but also were able to increase the levels of proinflammatory cytokines and chemokines, such as IL-12 and TNF-α, in the TME, thereby enhancing the antitumor immune response. A graphene quantum dot capable of forming a heterogeneous structure by combining with copper-based nanomaterials was prepared by Yan et al. and was able to promote the activation of immune cells directly or indirectly through its own properties, in addition to targeting and damaging DNA, thereby releasing tumor-associated antigens and promoting the maturation of DCs and the activation of T cells. In addition, selenium nanoparticles prepared by Xiong et al. (107) were able to promote ROS generation in tumor cells, leading to elevated levels of oxidative stress and thereby inhibiting tumor cell growth and proliferation. Xu et al. (108) synthesized PAP-SeNPs by integrating selenium nanoparticles with polysaccharides derived from Pholiota adiposa. Utilizing the diverse benefits of polysaccharides, including their degradability and biocompatibility, they improved the stability of selenium nanoparticles. These nanoparticles effectively targeted M2 TAMs and induced their polarization into the M1 phenotype, which exhibits anticancer properties. Li et al. (109) created a nanoplatform (Nano-Bi_2_Se_3_@MnCaP). This platform’s photodynamic treatment (PDT) can efficiently induce immunogenic cell death, stimulate the immune system, facilitate dendritic cell maturation, and activate T cells, therefore augmenting the antitumor immune response.

**Figure 9:**
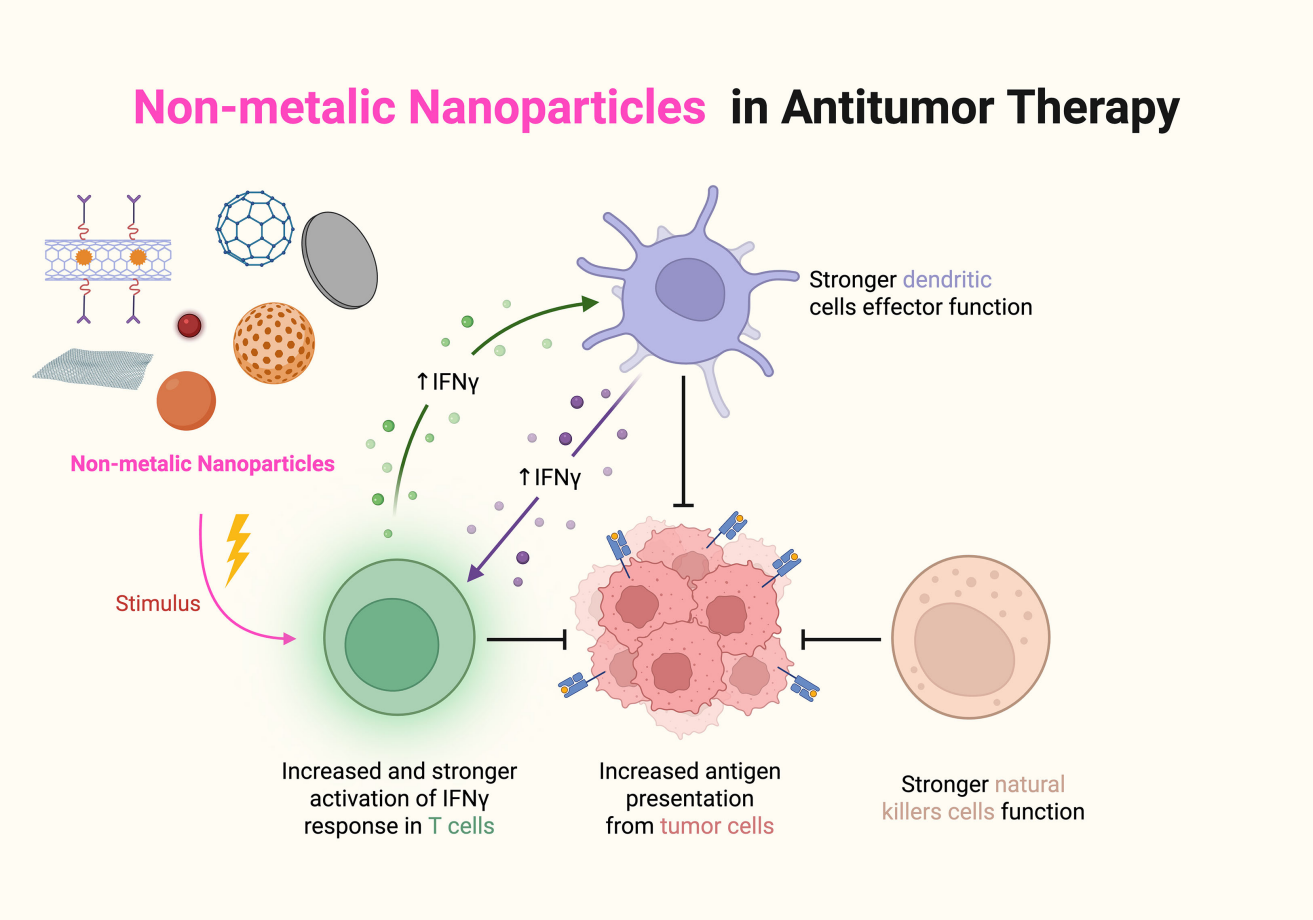
The effects of nonmetallic nanomaterials on the TIME.

Mesoporous silica nanoparticles have excellent drug-carrying capacity and good hydrophilicity and have great potential for development (110). Zhao et al. (111) loaded acidic, environment-responsive CCM-encapsulated mesoporous silica nanoparticles with dacarbazine and combined them with aPD1. These nanoparticles not only induced an antitumor immune response in T cells but also effectively inhibited the growth and metastasis of melanoma. Wang et al. (112) prepared hollow mesoporous silica (HMS) nanospheres, which were able to promote the maturation of DCs. In addition, the HMS cancer vaccine exerted a synergistic effect with anti-PD-L1 antibodies on the tumor, which effectively increased the levels of CD4^+^ and CD8^+^ T cells and ultimately inhibited the growth of tumors (see **Table 3**) (**Figure 9**).

**Table 3:** Functions and advantages of inorganic nanomaterials in the TIME.

Category	Name	Function in TIME	Advantages	References
Metal nanomaterials	Gold nanoparticles	Gold nanoparticles can carry immunomodulators photothermal therapy and photodynamic therapy	Good biocompatibility, low toxicity to normal cells Generate ROS and promote cell apoptosis Improve the stability of chemotherapeutic drugs	(142–144)
	Titanium dioxide nanoparticles	Induce mitochondrial dysfunction produced by ROS overload, significantly promote osteosarcoma cell apoptosis *in vitro*, and effectively inhibit tumor growth *in vivo*	Excellent anticancer properties and good biocompatibility	(145)
	Mesoporous nanoparticles	Regulate the phenotype of TAMs Promote the conversion of M2 type macrophages to M1 type	Low toxicity to normal tissue Enhanced osmotic and retention effects	(146)
Nonmetallic nanoparticles	Calcium hydroxide nanoparticles	The numbers of immunosuppressive cells were significantly reduced, while those of immunosupportive cells (e.g., M1-type macrophages and T cells) were significantly increased in nano-CaH2-treated tumors.Significantly inhibit the growth of primary and distant tumors	Ability to regulate pH Neoplastic growth was significantly curtailed, and overall survival was significantly augmented in the nano-CaH_2_-treated group	(147)
	CaCO_3_ nanoparticles	React with protons in the tumor to neutralize the acidic environment, thereby improving the tumor microenvironment Reduce the number of MDSCs and Tregs	Good biocompatibility, no significant tissue inflammation or damage seen in major organs pH-sensitive and able to enhance immune response	(148)

### 4.3 Hybrid nanomaterials

Hybrid nanomaterials refer to new materials formed by combining two or more different materials through a specific method. Gyu et al. (113) fabricated a composite nanomaterial of galactose-rich polysaccharides extracted from plant cell walls, which induced the conversion of M2-type tumor-associated macrophages to M1-type phenotypes, thereby enhancing the tumor immune response and mitigating the immunosuppressive TME. Gao et al. (114) used AMD3100-modified poly(lactic-coethanolic acid) nanoparticles as sorafenib carriers and prepared ADOPSor nanoparticles, which were shown to be able to effectively inhibit the infiltration of TAMs in hepatocellular carcinoma. Zhang et al. (115) prepared composite nanomaterials consisting of gold nanorods, manganese dioxide, and silicon dioxide, which enabled the loading of glucose oxidase; subsequently, they applied cancer cell starvation therapy and photothermal therapy to detect tumor cells; the results revealed that the material was not only able to increase the oxygen level in the TME but also to enrich more efficiently at the tumor site, thus improving the therapeutic effect.

Feng et al. (116) prepared a nanomaterial consisting of bacterial outer membrane vesicles combined with CD47 nanoantibodies; it not only specifically binds to CD47 molecules on the surface of tumor cells and Toll-like receptors on the surface of macrophages but also promotes the maturation of DCs as well as the recruitment of a variety of immune cells into tumor tissues, thus remodeling the TME. Chen et al. (117) loaded ovalbumin and copper sulfide into PLGA nanoparticles to form nanocomplexes with a core-shell structure and revealed that the nanomaterials were able to activate CD8^+^ T cells and induce a tumor immune response.

## 5 Clinical trials related to nanomaterials regulating the immune microenvironment

**Figure 10:**
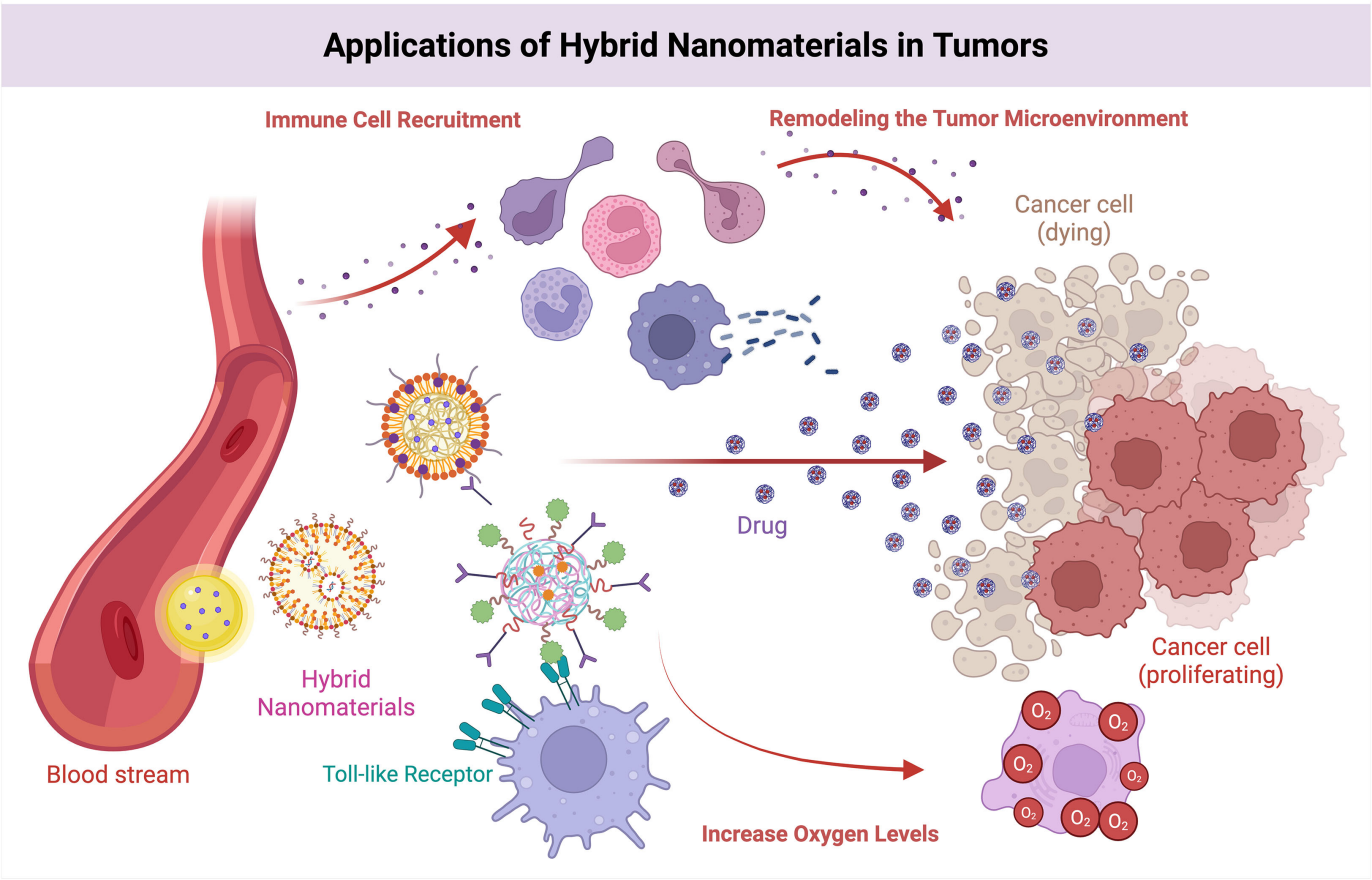
Effects of hybrid nanomaterials on the TIME. Hybrid nanomaterials stimulate the immune system via TLRs, facilitate targeted drug delivery to eradicate cancer cells, attract immune cells to modify the microenvironment.

Nanomaterials have demonstrated remarkable potential in influencing the tumor immune microenvironment and have subsequently become a hot topic in research (33). During the past few years, several clinical studies have been carried out to explore the application of nanomaterials in immunotherapy to improve its effects, including amplifying immune cell activity, the promotion of antigen presentation, and the alleviation of immunosuppression. Therefore, we collated the targets of different types of nanomaterials in immunotherapy and their ability to improve the tumor immune microenvironment (TIME) (see **Table 4**) (**Figure 10**).

**Table 4:** Different types of nanomaterials used in tumor immunotherapy.

Targets	Material type	Cancer modeling	Conclusion	References
Tregs	PEG-modified single-walled carbon nanotubes	B16 Melanoma	Selectively targets Tregs in tumors and enhances tumor vascular permeability Increases CD8+ T cells in C57BL/6 mice	(149)
	Hybrid nanoparticles (tLyp1-hNPs)	DU145/B16 mouse model	Hybrid nanoparticles target Tregs and combine with immune checkpoint blockers (e.g. anti-CTLA-4 antibodies) to enhance anti-tumor immune responses and inhibit Treg cell differentiation and proliferation	(150)
	Organic complex nanoparticles (PTEQ nanoparticles)	CT26 cells	PTEQ nanoparticles promote maturation of DCs and polarization of M1-type macrophages and enhance anti-tumor immune responses	(83)
	Polymer nanoparticles PIMDQ/Syro-NP	4T1 breast cancer cells	Inhibits proliferation and function of Tregs, activates DCs and promotes their maturation and function, and enhances infiltration and activity of effector T cells	(151)
TAMs	Cyclodextrin nanoparticles	MC38 colorectal tumor cells	Enhancement of T-cell effector function	(152)
	Polymer nanoparticles	4T1 breast cancer cells	Nanomaterial-treated tumor cells release mtDNA fragments that are taken up by TAMs, activate the cGAS-STING pathway, and undergo reprogramming, which helps to alleviate immunosuppression in the tumor microenvironment and enhances T-cell-mediated anti-tumor immune responses	(153)
	Bionic Nanocarriers	4T1 tumor model	Activation of the TLR7/8 signaling pathway in TAMs induces conversion from an immunosuppressed M2 to M1 type; secretion of pro-inflammatory cytokines and activation of immune responses.	(154)
	Gene editing nanoparticles	B16F10 melanoma cells	Promote phagocytosis of cancer cells by macrophages and enhance T-cell-mediated anti-tumor immune responses by improving antigen presentation	(155)
MDSCs	Nanocage containing gemcitabine	Mouse model of triple-negative breast cancer	Significantly reduces the proportion of MDSC while increasing T-cell infiltration.	(156)
	Lipid-coated biodegradable hollow mesoporous silica nanoparticles	B16 F10 Homozygous C57/BL 6 Mouse	Nanoparticle-mediated combination therapies can benignly modulate the tumor microenvironment through the activation of tumor-infiltrating T lymphocytes and natural killer cells, as well as the promotion of IFN-γ and IL-12 cytokine secretion.	(157)

## 6 Summary and discussion

The tumor microenvironment is a highly complex ecosystem comprising a diverse array of cell types, including immune cells, as well as various cytokines, chemokines, and signaling molecules. The key cell types include mainly macrophages, tumor-associated fibroblasts, myeloid-derived suppressor cells (MDSCs), dendritic cells, natural killer cells and effector T cells; of these, macrophages usually tend to be protumorigenic with an M2 phenotype, promoting tumor growth and angiogenesis (118), whereas regulatory T cells and MDSCs promote immunosuppression through the inhibition of effector T cells and natural killer (NK) cells, thus creating a supportive tumor environment for tumor development (119). The immunotherapy of tumors often involves these cytokines; therefore, targeting these cytokines can improve therapeutic efficacy.

Methods for targeting the TME to address this problem have been widely investigated; however, improving therapeutic efficacy more efficiently and rapidly is particularly important. Although conventional treatments are effective against tumors, they still have some shortcomings. Nanomaterials have made great progress as a novel means (92), and the rise of nanomaterials not only improves therapeutic efficacy but also improves precision and reduces toxic side effects (120). Owing to the unique physical and chemical properties of nanomaterials, the infiltration and activation of immune cells can be effectively enhanced to improve antitumor immune responses. This paper summarizes the main mechanisms by which different types of nanomaterials modulate the TME. Nanomaterials can not only deliver immune checkpoint inhibitors, such as anti-CD47 antibodies but also enhance the ability of immune cells to kill tumor cells (121). In addition, nanomaterials can be combined with a variety of therapies based on graphene materials, such as graphene oxide-polyethylene glycol-polyethyleneimine-CpG nanocomplexes loaded with CpG ODN, which utilize the near-infrared light absorption of graphene oxide, thus enhancing intracellular delivery and achieving a perfect combination of photothermal and immunotherapy (122).

Despite the good biocompatibility of nanomaterials, further long-term assessments of their toxicity in living organisms and clinical trials are needed. The main limitations of currently employed immunotherapy strategies are the inability of most immunotherapeutic drugs to reactivate T cells and expand them *in vitro* or *in vivo* and the various complicating factors that lead to T-cell exhaustion in the context of tumor–TME interactions and the acquisition of immune resistance (33). In addition, the clearance mechanism of nanomaterials *in vivo* has not yet been clarified, and in the future, we can focus on their retention *in vivo* and whether they affect organ function. Finally, the drug delivery rate of nanomaterials still needs to be optimized, and owing to the complexity of the TME, the distribution of nanomaterials in tumor tissues is still limited, even though some drugs can be delivered effectively to the intended targets by nanomaterials. Emerging technologies, particularly artificial intelligence, possess significant potential. They are not only efficient and precise but also possess the capability to forecast therapy outcomes. In the future, further developing technologies may be incorporated with nanomaterials to augment medicinal effects.

In summary, nanomaterials have potential in regulating the TIME and offer new therapeutic tools for the immunotherapy of tumors. However, issues such as the biotoxicity and drug delivery rate of nanomaterials still need to be further explored. Therefore, in the future, more efficient and safer nanomaterials could be translated into the clinic, and multidisciplinary intersections could be applied to provide patients with more precise and effective therapeutic options.
